# Bodily Reactions to Emotional Words Referring to Own versus Other People’s Emotions

**DOI:** 10.3389/fpsyg.2017.01277

**Published:** 2017-08-22

**Authors:** Patrick P. Weis, Cornelia Herbert

**Affiliations:** ^1^Department of Psychiatry, University of Tübingen Tübingen, Germany; ^2^Institute of Psychology and Education, Applied Emotion and Motivation Research, University of Ulm Ulm, Germany

**Keywords:** emotion, self, language, embodiment, facial expression, heart rate, skin conductance, emotional communication

## Abstract

According to embodiment theories, language and emotion affect each other. In line with this, several previous studies investigated changes in bodily responses including facial expressions, heart rate or skin conductance during affective evaluation of emotional words and sentences. This study investigates the embodiment of emotional word processing from a social perspective by experimentally manipulating the emotional valence of a word and its personal reference. Stimuli consisted of pronoun-noun pairs, i.e., positive, negative, and neutral nouns paired with possessive pronouns of the first or the third person (“my,” “his”) or the non-referential negation term (“no”) as controls. Participants had to quickly evaluate the word pairs by key presses as either positive, negative, or neutral, depending on the subjective feelings they elicit. Hereafter, they elaborated the intensity of the feeling on a non-verbal scale from 1 (very unpleasant) to 9 (very pleasant). Facial expressions (*M. Zygomaticus*, *M. Corrugator*), heart rate, and, for exploratory purposes, skin conductance were recorded continuously during the spontaneous and elaborate evaluation tasks. Positive pronoun-noun phrases were responded to the quickest and judged more often as positive when they were self-related, i.e., related to the reader’s self (e.g., “my happiness,” “my joy”) than when related to the self of a virtual other (e.g., “his happiness,” “his joy”), suggesting a self-positivity bias in the emotional evaluation of word stimuli. Physiologically, evaluation of emotional, unlike neutral pronoun-noun pairs initially elicited an increase in mean heart rate irrespective of stimulus reference. Changes in facial muscle activity, *M. Zygomaticus* in particular, were most pronounced during spontaneous evaluation of positive other-related pronoun-noun phrases in line with theoretical assumptions that facial expressions are socially embedded even in situation where no real communication partner is present. Taken together, the present results confirm and extend the embodiment hypothesis of language by showing that bodily signals can be differently pronounced during emotional evaluation of self- and other-related emotional words.

## Introduction

Theoretical considerations have long been emphasizing the independence of language and emotion. Semantic network models of language, for instance, consider language mainly as a cognitive phenomenon, its representation bearing no direct relation to sensory, sensorimotor or affective processes in the brain or the body (e.g., Semin and Smith, 2008; or Winkielman et al., 2015 for an overview). However, neurophysiologic research has proven otherwise: language and emotion processing affect each other. This has been shown for the processing of simple words (e.g., Martín-Loeches et al., 2001; Tabert et al., 2001; Kuchinke et al., 2005; Kissler et al., 2007; Herbert et al., 2008, 2009; Scott et al., 2009) and sentences (e.g., Bayer et al., 2010; Jiménez-Ortega et al., 2012). Regarding the processing of single words, rapid serial presentation of words with emotional content, in several studies, facilitated both initial stimulus processing in the visual cortex and subsequent recall performance of emotional words (e.g., Kissler et al., 2007; Herbert et al., 2008). Moreover, in several studies, reading emotional words activated emotional brain structures such as the amygdala (Hamann, 2001; Tabert et al., 2001; Kuchinke et al., 2005; Hazlett et al., 2007; Herbert et al., 2009) and induced changes in affective behavior including priming of approach and avoidance including defensive responses like the startle-reflex (e.g., Herbert et al., 2006; Herbert and Kissler, 2010; Citron et al., 2016). Presentation of emotional words also influences the perception and appraisal of non-verbal emotional signals: on a behavioral (e.g., Lindquist et al., 2006) as well as on a neural or physiological level (Lieberman et al., 2007; Moseley et al., 2012; Herbert et al., 2013a,c), having implications for the treatment of clinical and neurological disorders (Roberson et al., 2007; Kircanski et al., 2012).

Thus, preferential processing of emotional words, activation of emotional brain structures as well as changes in affective behavior during word processing could be taken as evidence for theories of embodiment arguing that written language is able to elicit, modulate and regulate emotional processes in the brain and the body (see Niedenthal, 2007; Glenberg et al., 2009).

This, however, raises the question about the social relevance of embodied language processing. Expressing one’s own emotions to others as well as inducing emotions in others is a key function of spoken and written language. This holds true even in situations in which no direct face-to-face communication is possible: for instance, we text, blog, and tweet our sentiments to others and “like/dislike” others for their affection. But to what extent is language processing embodied when we assess and appraise emotional content related to one’s own self (e.g., “my fear”) or the self of another person (e.g., “his fear”), especially in contexts and situations where input from non-verbal modalities is not readily available to the perceiver of the message? In other words, will bodily, peripheral-physiological reactions differ as a function of the valence or as a function of the self-other reference of a word? Crucially, what does this mean theoretically for the embodiment of language and more generally for the embodiment of emotional communication?

Regarding emotional communication, the human face has been considered an important “socio-emotional signal detector,” even in the absence of direct face-to-face communication (e.g., Fridlund, 1991; Buck, 1994; Hess et al., 1995). Whether facial expressions are, however, more important for understanding one’s own rather than other people’s emotions is still under scientific debate. For instance, Hess et al. (1992) could demonstrate that spontaneous elicitation of facial expressions influences primarily the perception of one’s own subjective emotional experiences. Other studies found that people spontaneously mimic other people’s emotional expressions (Chartrand and Bargh, 1999), even if the other is only imagined as a virtual other (Fridlund, 1991). In these latter views, spontaneous simulations of emotions via facial expressions are not just reflexive readouts of one’s own emotions (e.g., Buck, 1994 for an overview) but may preferentially occur in response to other-related emotional stimuli, in particular to positive stimuli (Fridlund, 1991). One purpose of this “sociality effect” (Fridlund, 1991) could be to help evaluate the hedonic quality of other-related stimuli by using one’s own facial expressions as proxy.

Regarding language processing, involvement of facial expressions has been reported in several recent studies recording facial muscle activity during emotional evaluation of words and sentences (electromyography, EMG; e.g., Foroni and Semin, 2009; Niedenthal et al., 2009; Havas et al., 2010). These studies revealed that reading positive words or sentences is accompanied by activation of the main facial muscle used for smiling, *M. Zygomaticus*, whereas reading words, sentences, or statements with negative content is accompanied by activation of the main facial muscle used for frowning, *M. Corrugator* (see also Foroni and Semin, 2009; Niedenthal et al., 2009; Foroni and Semin, 2011). Moreover, negating the emotional meaning of a positive statement has been found to be associated with attenuated *M. Zygomaticus* activity (Foroni and Semin, 2013), suggesting that changes in facial expressions during emotional word processing are related to semantic processing and word comprehension. This is also suggested by recent observations about physiological or experimental manipulation of facial muscle activity, including studies on facial Botox treatment or suppression of the facial musculature by holding a pen with the lips or teeth indicating that inhibiting facial expressions impairs specifically the comprehension (Havas et al., 2010) and emotional evaluation of emotional statements (Niedenthal et al., 2009; also see Strack et al., 1988 using cartoons). In addition, changes in facial muscle activity have been found to be more pronounced in language tasks affording emotional instead of cognitive evaluation (Niedenthal et al., 2009) and for concrete compared to abstract emotional words (Foroni and Semin, 2009, 2013), although, overall, changes in facial muscle activity seem to be less pronounced for written words than for pictures or scenes (Larsen et al., 2003), probably due to the lower arousal of word as compared to picture stimuli.

Taken together, the aforementioned findings support the idea of facial expressions being paramount for the decoding and appraisal of the emotional meaning of language stimuli. However, previous language studies have not considered the influence social factors may have on emotional language processing, leaving open the theoretical question of whether participants will be mimicking more during emotional evaluation of other-related than self-related emotional words, or vice versa.

Regarding the perception of one’s own emotions, historically (see e.g., Sorabji, 1992; Höffe et al., 2005) and metaphorically, the heart has been proposed as the central core of one’s own feelings. In fact, individuals who are able to accurately detect their own heart beats experience emotions with heightened intensity (Wiens et al., 2000). They also seem to intuitively make use of their cardio-visceral reactions for decision making (Dunn et al., 2006; Werner et al., 2009) although this does not always promote favorable decisions (Dunn et al., 2010). Additionally, changes in cardiac cycle as well as in parasympathetic tone, as measured by heart rate variability (HRV), can influence social cognition, emotional stimulus processing, and later semantic memory retrieval (Wallentin et al., 2011; Quintana et al., 2012; Garfinkel et al., 2013). Even though these studies do show that interactions between emotional, mental, and cognitive processing are accompanied by cardiac changes, regarding emotion and language processing, only a few studies have investigated stimulus-driven changes in mean HR during processing of emotional words. The studies available used a mix of spoken or written (synthesized) words, sentences, and stories, in combination with autobiographical imagery, recall, or cognitive instructions (Vrana et al., 1986; Ilves and Surakka, 2012), or presented highly selective self-relevant stimulus materials such as threat words or body words to particular samples of individuals at risk for anxiety (Thayer et al., 2000) or eating disorders (Herbert et al., 2013b), impeding the generalizability of the results.

Crucially, with a few exceptions, previous studies did not explicitly control for the words’ personal reference, i.e., whether the emotional content of a word was related to the reader’s own self or the self of another person. In an earlier study by Cacioppo et al. (1985), positive and negative trait adjectives were presented to healthy students who were asked to judge each word according to orthographic and grammatical rules, or to evaluate the words for hedonic pleasure (“is this word good?”) and self-descriptiveness (“does this trait describe you?”). Mean HR differed during semantic (emotional and self-related) and non-semantic (orthographic and grammatical) evaluation. However, mean HR did not differ significantly between the emotional and self-referential evaluation tasks, suggesting no specific influence of the self-relatedness of the task on changes in HR during word processing. This observation contrasts with findings from text-driven imagery where often considerably strong HR acceleration patterns were reported during imagery of autobiographic, self-related emotional scenes (Vrana and Rollock, 2002). Thus, so far, no clear picture has emerged with regard to whether HR varies as a function of the personal reference of a word (i.e., self- vs. other-reference), or whether during word processing changes in HR indicate differences in emotional content (positive, negative, or neutral) and depth of stimulus elaboration (e.g., Cacioppo et al., 1985), regardless of the word’s personal reference.

Regarding neurophysiological processes in the brain, self-reference seems to be uniquely linked to emotional processing (e.g., Northoff et al., 2006; D’Argembeau et al., 2012). Regarding verbal stimuli (e.g., Esslen et al., 2008; Herbert et al., 2011c), there is evidence that processing of emotional words related to the reader’s self increases activity in anterior cortical midline structures (medial prefrontal cortex, including the anterior cingulate cortex and the ventromedial prefrontal cortex), i.e., brain structures involved in self-referential processing of emotional stimuli (Northoff et al., 2006 for an overview). In addition, electrophysiological studies reported sustained cortical processing and better free recall performance of especially self-related positive words (Watson et al., 2007; Herbert et al., 2011b). Mood congruent processing has been proposed as the possible underpinning of this prioritized processing of positive stimuli related to the self; mildly positive mood being the norm in healthy Western subjects (see Herbert et al., 2011d for modulation with depression; Mezulis et al., 2004; Shi et al., 2016 for cross-cultural findings; Taylor and Brown, 1988).

In view of the observations outlined above, the present study’s aims are to contribute to the so far fragmentary understanding of self-other reference and bodily involvement in language and emotion processing. To this end, a novel paradigm (see Herbert et al., 2011b,c) is deployed to investigate peripheral physiological responses to self- and other-related words with emotional and neutral content. Unlike many previous studies summarized above, in the present paradigm, the emotional valence and the personal reference of a word are altered simultaneously by using pronoun-noun pairs that are related to the reader’s self (e.g., “my fear,” “my joy”) or other-related, i.e., related to the self of a virtual other (e.g., “his fear,” “his joy”). Physiological responses are measured while participants read and quickly judge the pronoun-noun phrases for hedonic pleasure/displeasure and then evaluate them with respect to the intensity of their subjective feelings. An additional set of stimuli consisting of negated emotional and neutral words (e.g., “no fear,” “no happiness,” or “no book”) is included as a control condition to determine whether participants’ spontaneous judgments and their initial physiological reactions will be based on the evaluation of the words’ semantic meaning as proposed by previous research (e.g., Foroni and Semin, 2013). The physiological measures include recording facial muscle activity (fEMG), HR, and skin conductance (electrodermal activity, EDA), the latter being included for exploratory purposes to control for physiological arousal.

Extending previous research, the following questions are addressed: How does self- versus other-reference influence emotional word processing on a behavioral, subjective and peripheral-physiological level? Is the processing of self-related positive words prioritized on a behavioral level indicating better access to one’s own positive emotions in healthy subjects? Is this preference also reflected at a physiological level and associated with changes of fEMG or HR? In particular, do participants respond with differential fEMG to emotional words depending on whether the emotional content is self- or other- related? Lastly, is HR variation during emotional word evaluation sensitive to the emotional valence of a word, the self-reference of a word, or both?

## Materials and Methods

### Participants

In total, twenty-nine young healthy adults (five males, *M* = 22.8 years, *SD* = 2.6; range: 18–28 years), all students of the University of Tübingen, native speakers of German, with normal or corrected to normal vision, and normal depression scores (see **Table 1** for an overview) were included in the study. Twenty-eight subjects were non-smokers and one subject reported occasional smoking with less than half a cigarette a day. Caffeine intake was controlled at the day of testing. In addition, habitual drinking habits were assessed by self-report scales. Three subjects were left-handed. Participants were to report that they are currently taking no medication that might affect emotional functioning or interact with the acetylcholinergic system. They provided written informed consent prior to participation and were compensated with an hourly wage of eight Euros in return for participation. The study was approved by the local Ethics Committee (https://www.medizin.uni-tuebingen.de/Forschung/Ethik\_Kommission.html).

**Table 1:** Descriptive statistics of questionnaire data (*N* = 29).

Statistic	Questionnaire						
	
	BDI-II	TAS20	PANAS NA	PANAS PA	MWT-B	STAI state	STAI trait
Mean	4.8	41.1	11.9	28.5	27.3	35.2	36.3
(SD)	(3.6)	(8.6)	(3.0)	(6.6)	(3.6)	(6.9)	(8.0)
Range	0–11	25–60	9–23	18–40	19–33	24–49	24–55


*Means, standard deviations (SD), and ranges are reported. For explanations of abbreviations and more information on the questionnaires, see procedure.*

### Procedure

Participants were familiarized with the laboratory setting and the experiment was explained to them in general terms before giving informed consent. Participants were asked about social demographics and handedness (German version of Oldfield, 1971), and received written instructions. In particular, participants were instructed that words paired with the possessive pronoun of the third person “his” are related to a virtual other whereas words paired with the possessive pronoun of the first person “my” are related to themselves, e.g., describing the reader’s own emotions. Participants received practice trials and had to repeat the instructions to the experimenter in their own words prior to the start of the experiment to ensure that they had understood the instructions. The main experiment, following the practice trials lasted approximately 60 min. After experimental testing participants completed questionnaires as described subsequently. The state scale of the Positive and Negative Affect Schedule (PANAS state; Watson et al., 1988) and the Beck Depression Inventory II (BDI-II; Hautzinger et al., 2006) were administered to control for mood effects and possible risk for depression. The State-Trait Anxiety Inventory (STAI; Laux et al., 1981) was used to control for state and trait anxiety. The Toronto Alexithymia Scale (TAS-20; Bagby et al., 1994) enables identification of alexithymic individuals who should only have attenuated access to their evoked feelings. The Mehrfachwahl-Wortschatz-Intelligenztest (MWT-B, a verbal IQ test; Lehrl, 2005) allows quantification of familiarity with German language which is relevant for correct processing of the presented stimulus material. Although self-report data was assessed primarily to exclude participants scoring high on alexithymia, depression or anxiety, alexithymia, depression and anxiety scores as well as scores of positive and negative affect were later on also used in exploratory analyses assessing potential interindividual differences in behavioral and physiological measures. At the very end, subjects were asked about potential strategies they might have used during the main experiment and were debriefed if desired.

### Experimental Design

Stimuli were presented on a computer screen. Participants’ task was to read the words silently and to spontaneously judge the words for hedonic pleasure/displeasure (i.e., “is this word eliciting a positive, negative, or neutral feeling?”) before evaluating each word in detail with respect to the intensity of the subjectively experienced feeling (i.e., “how intense is the feeling elicited by the word?”). Participants were instructed to base their judgments solely on their gut feelings and decide as quickly and as spontaneously as possible. Spontaneous judgments included a quick button press for a coarse valence judgment (negative, neutral, or positive) for which participants had to press one of three keyboard buttons. The response assignment to keys was counterbalanced across participants with the middle button remaining the neutral response for all participants and the left and right buttons altering in response assignment between ‘negative/unpleasant’ and ‘positive/pleasant.’ The subsequent elaborate evaluation, following the spontaneous judgment, included a voice response. For the voice response, the valence scale of the nine-point self-assessment manikin (SAM; Lang, 1980) was presented to participants before the start of the experiment, to familiarize them with the scale, and also after each stimulus block during the experiment as reminder. Participants were told to evaluate the intensity of their stimulus-evoked feelings by naming a number corresponding to the manikin that fits best to the evoked feeling. Number assignments always started with ‘1’ at the outermost left manikin counting up to ‘9’ at the right outermost manikin.

**FIGURE 1:**
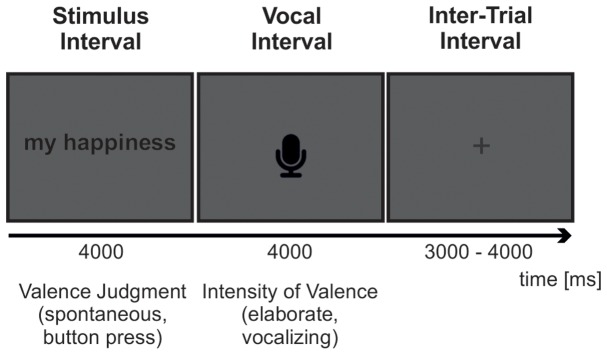
Trial Sequence. Each trial consisted of a 4000 ms stimulus interval followed by a 4000 ms vocal response interval and an inter-trial interval uniformly distributed between 3000 and 4000 ms. Subjects had to coarsely judge the pronoun-noun phrases in reference to their feelings using one of three keyboard buttons (spontaneous valence judgments). The button press had to occur during the 4000 ms time window of stimulus presentation. As soon as the microphone icon appeared, subjects had to elaborate the intensity of the evoked feelings by speaking out the numbers ‘one’ to ‘nine’ corresponding to the nine-point self assessment manikin scale of emotional valence (elaborate valence judgment).

Each trial started with the presentation of a pronoun-noun pair. The pair was presented in upper case in the middle of the computer screen for 4000 ms. The button response had to be given while the stimulus remained at the display. Subsequently, a microphone icon was presented for 4000 ms indicating the voice response interval, in which participants were asked to elaborate the intensity of the feeling elicited by the stimulus during the spontaneous appraisal. Interstimulus intervals were uniformly distributed between 3000 and 4000 ms. An overview of the experimental task is provided in **Figure 1**.

### Stimulus Material and Stimulus Matching

Each stimulus consisted of one out of three different pronouns (“mein/e,” German for “my,” “sein/e,” German for “his,” or “kein/e,” German for “no”) and one out of 84 nouns categorized into three different valence categories (negative, neutral, or positive), resulting in a 3^∗^3 (reference^∗^valence) design. Nouns were used for valence manipulation; pronouns were used for reference manipulation (“my” for self-reference, “his” for other-reference, and “no” for no reference). Only the male version of the third person German possessive pronoun was used for other-reference because in German language the female version of the third person possessive pronoun could be ambiguous (referring either to “her” or “their”). Each of the 84 nouns was paired with all possible pronouns (e.g., “my fear,” “his fear,” and “no fear”), resulting in 252 trials in total. Trials were presented in blocks, each block consisting of four trials with the same pronoun and the same valence category. A randomized block-design was chosen in order to avoid changes in physiology due to an increase in cognitive load or mental effort which might have been likely to occur when switching one’s evaluation from trial to trial. Therefore, pronoun order was randomized while the following rule was considered: within three consecutive blocks, each pronoun (self-related, other-related, or no-reference) was used once and there were no adjacent blocks with the same pronoun. Valence order was randomized while the following rule was considered: within every nine blocks, every noun valence category was paired with every pronoun exactly once and adjacent blocks never had the same valence.

Nouns were taken from the German affective word list BAWL-R (Võ et al., 2009) and were matched on several dimensions including stimulus valence (*-3: very negative to 3: very positive*), arousal (*1: low arousal* to *5: high arousal)*, imageability (*1: low imageability* to *7: high imageability*), and total frequency of appearance per million words (FTOT). Out of the over 800 nouns included in the BAWL-R with either negative (valence < -1.5), neutral (0.2 ≥ valence ≥-0.2) or positive (valence > 1.5) valence, 28 were selected for each emotional category. The selection procedure was based on matching between valence groups for arousal, FTOT, word length, gender, and compatibility with the used pronouns. To control for compatibility effects between pronouns and nouns, we assessed whether the respective pronouns occurred as significant left occurrences of each of the 84 nouns in our list. For this procedure, a German linguistic corpus (Wortschatz Universität Leipzig^1^) was used. The procedure used for extracting significant left occurrences within the *Wortschatz Universität Leipzig* was described by Biemann et al. (2004).

Positive, negative, and neutral nouns differed significantly in arousal, *F*(2,81) = 38.66, *p* < 0.001. There was no difference in arousal between positive and negative nouns, *T*(54) = 0.25, *p* = 0.81, but between positive and neutral, *T*(54) = 6.86, *p* < 0.001, and between negative and neutral, *T*(54) = 7.90, *p* < 0.001, nouns. There was no difference in word length, *F*(2,81) = 0.04, *p* = 0.96, FTOT, *F*(2,81) < 0.01, *p* > 0.99, or imageability, *F*(2,81) = 2.16, *p* = 0.12, between valence groups. The clustering into different valence categories was successful, *F*(2,81) = 18.62, *p* < 0.001. Negative nouns differed in valence from neutral, *T*(54) = 3.49, *p* = 0.002, and positive, *T*(54) = 4.98, *p* < 0.001, nouns. Positive nouns differed in valence from neutral nouns, *T*(54) = 3.59, *p* = 0.001. Means and standard deviations of all parameters, depending on valence group, are summarized in **Table 2**.

**Table 2:** Descriptive statistics of noun stimuli parameters.

Parameter	Valence		
	
	Negative	Neutral	Positive
Arousal	3.48 (0.36)	2.51 (0.53)	3.45 (0.49)
FTOT	45.38 (65.00)	44.57 (64.27)	45.17 (62.85)
Letters	6.64 (1.59)	6.54 (1.55)	6.54 (1.55)
Imageability	3.59 (1.13)	4.12 (1.29)	4.19 (1.12)
Valence	-1.86 (0.26)	-0.01 (0.09)	1.92 (0.29)
Arousal	3.48 (0.36)	2.51 (0.53)	3.45 (0.49)
FTOT	45.38 (65.00)	44.57 (64.27)	45.17 (62.85)


### Apparatus and Recording

Visual stimuli were presented at a distance of 100 cm on a 22-inch monitor (Eizo SX2262W, 1650 × 1024 pixel resolution, 60 Hz frame rate) using MATLAB version R2011a (The Mathworks, Inc., Natick, MA, United States) and the Psychophysics Toolbox (Brainard, 1997; Pelli, 1997). Button press responses were recorded using a PS/2 connected standard keyboard. Voice responses were recorded using a standard interfacial microphone (Samson Technologies, Hauppauge, NY, United States). Physiological data, i.e., data from electrocardiogram (ECG), EDA, and fEMG at *M. Corrugator* and *M. Zygomaticus* regions, was registered using the mobile Varioport amplifier (Becker Meditec, Karlsruhe, Germany), which allows data sampling at a rate of 512 Hz. The EMG electrodes were placed in accordance with methodological guidelines (Fridlund and Cacioppo, 1986). For electrocardiography, one-way electrodes were used which were placed at the upper sternum, lower sternum, and left lateral margin of the chest. This placement is thought to lead to minimal movement artifacts (Jennings et al., 1981). Skin conductance including EDA was registered using direct current stimulation with Ag/AgCl electrodes of a diameter of 4 mm. Due to hardwired settings of the amplifier, the physiological signals were filtered online. The ECG-signal was band-pass filtered at 0.9–100 Hz (-3 db). The fEMG-signal was band-pass filtered at 70–400 Hz (-3 db). While the attenuation of the fEMG-signal in the low frequency range leads to a reduction of power line noise, it also decreases a sizable part of the surface EMG signal which has most of the energy between 10 and 200 Hz (Tassinary et al., 2007). Usage of the 70 to 400 Hz passband attenuates especially weak signals originating from single motor unit firing as opposed to aggregated motor unit activity (Tassinary et al., 2007). The usage of the described online filter may thus lead to a distortion of the fEMG-signal, especially when interested in periods of low fEMG, which, however, was not analyzed in the present study.

### Preprocessing of Behavioral and Physiological Data

Only reaction times in the time window of 100 to 3900 ms after stimulus onset were considered. Trials with multiple key presses were excluded from analysis resulting on average in a loss of 1.3–2% of trials per stimulus condition. Key presses related to participants’ spontaneous evaluations of the stimuli were coded offline from -1 (negative) to 0 (neutral) to +1 (positive). Participants’ elaborate evaluations were coded offline in line with the SAM from 1 to 9 (1: very unpleasant, 9: very pleasant).

Electromyography raw data was high-pass filtered to reduce movement and blink artifacts and subsequently full-wave rectified. Continuous data was visually inspected and epochs with remaining artifacts were rejected. On average, 5.1% of trials per condition had to be rejected. Afterwards, data was segmented into epochs from -1000 ms to 11000 ms with relation to stimulus onset and 1000 ms pre-stimulus baseline-correction was performed.

R-spikes in the raw ECG signal were extracted using the QRStool (Allen et al., 2007) and raw ECG data was inspected for artifacts. Between 9.8 and 13.5% of the trials were rejected in each of the nine conditions, suggesting a uniform distribution of rejections. After artifact rejection, each condition for each individual subjects included more than 70% of trials. Data was segmented into epochs starting 1000 ms before stimulus onset until 11000 ms after stimulus onset. Epochs were baseline-corrected using the 1000 ms interval before stimulus onset. The epoch length was chosen to fit the maximal length of a trial with the shortest jittering possible (i.e., 4000 ms stimulus interval, 4000 ms voice response interval, and 3000 ms inter-trial interval).

Electrodermal activity was visually inspected and epochs with noisy baseline or activity that could not be classified as stimulus-elicited SCR with regard to the criteria described by Boucsein (2012) were rejected. Afterward, data was segmented into epochs from -1000 ms to 11000 ms with relation to stimulus onset and 1000 ms pre-stimulus baseline-correction was performed. Data of eight subjects showed no reliable responses time locked to the onset of the stimulus (non-responders). Conservative inspection of artifacts resulted in the rejection of another eight subjects, such that finally only a sample size of 13 subjects was included into the analysis. Therefore, the interpretation of the exploratory EDA data may have only limited validity and generalizability and results from preliminary EDA analysis are reported only in the Supplementary Material.

### Manipulation Check

After the experiment participants were asked for potential processing strategies, which revealed no differences, confirming that all participants followed the instruction given. In particular, all participants stated that they used similar encoding or appraisal strategies for words with self-related, other-related and negated pronouns.

### Data Analysis: Behavior and Physiology

Arithmetic means of reaction times, spontaneous judgments (given via key press), and elaborate judgments (given via voice) were analyzed in separate repeated measurement analyses of variance (ANOVAs) using the factors *reference* (self-reference, other-reference, no reference) and *valence* (positive, negative, neutral). Participants’ key presses were coded as -1 (if associated with a ‘negative/unpleasant’ response), 0 (if associated with a ‘neutral’ response), and +1 (if associated with a ‘positive/pleasant’ response) and, to obtain index scores of response accuracy (ranging from -1 to +1), individual responses (positive, negative, or neutral key presses) were averaged for each word category separately^2^. Dependent *t*-tests were conducted as *post hoc* tests.

Changes in mean *M. Corrugator* and mean *M. Zygomaticus* activity (fEMG), HR, and EDA were statistically analyzed with repeated measures ANOVAs. For fEMG and HR, activity between 0 and 4000 ms after stimulus onset was analyzed. EDA activity was analyzed between 0 and 11000 ms after stimulus onset. The longer analysis window is due to the slow reactivity of this measure, i.e., changes in mean skin conductance are characterized by slow wave drifts lasting about 10 up to 16 s.

All physiological signals were also assessed across time to determine stimulus-locked fluctuations across the whole stimulus presentation period from 0 to 4000 ms. To this end, the continuously recorded ECG and fEMG-signals were clustered into time bins of 500 ms; EDA data was clustered into 1000 ms bins and analyzed from 0 to 11000 ms after stimulus onset. Ultimately, each repeated measures ANOVA contained the factors *reference* (self-related, other-related, negated), *valence* (pleasant, unpleasant, neutral valence), and *time*. The results of the time series analyses are reported in the Supplementary Material (beneath the respective time × amplitude plots in Supplementary Figures 1B, 2B, 3B, 5B).

Analyses of variance results are reported Greenhouse-Geisser corrected where appropriate. Significant main and interaction effects were further analyzed by dependent *t*-tests. *P*-values of *post hoc* tests were controlled for multiple comparisons according to the procedure suggested by Benjamini and Hochberg (1995) which controls for false discovery rate (FDR).

## Results

### Reaction Times

**FIGURE 2:**
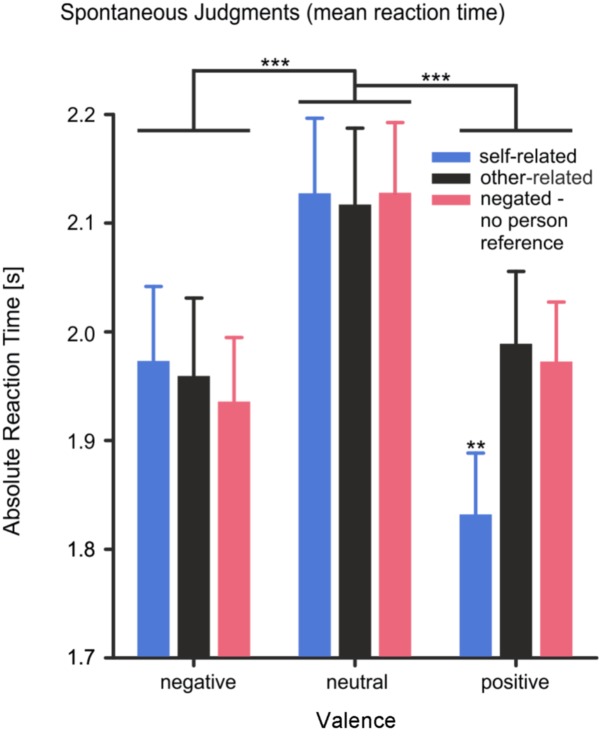
Mean reaction times of spontaneous judgments (absolute values). The reaction time represents the time subjects needed to spontaneously judge the valence of the pronoun-noun pairs. Error bars depict SEM. ^∗∗^*p* < 0.01, ^∗∗∗^*p* < 0.001, FDR corrected.

Reaction time showed a main effect of *valence*, *F*(2,56) = 18.12, *p* < 0.001, η^2^ = 0.39. Reaction times were significantly shorter for positive and negative words than for neutral words [negative vs. neutral: *T*(28) = -4.18, *p* < 0.001; positive vs. neutral: *T*(28) = -5.20, *p* < 0.001]. The main effect of *reference* was not significant, *F*(2,56) = 2.47, *p* = 0.094, η^2^ = 0.08. However, a significant interaction effect of *valence* × *reference*, *F*(4,112) = 5.62, *p* < 0.001, η^2^ = 0.17, was observed, supporting the hypothesis of a self-positivity bias. As shown in **Figure 2**, participants responded to self-related positive words significantly faster than to self-related negative or self-related neutral words and significantly faster than to other-related positive words [all three comparisons |*T*(28)| > 2.8, *p* < 0.01]. For self- and other-related negative or neutral words no difference in reaction times was found. Moreover, negated words without any personal reference were not responded to slower than other-related words [other-related vs. negated: *T*(28) = 0.405, *p* = 0.689], suggesting no considerable increase in task difficulty for the evaluation of negated compared to other-related stimuli.

### Judgments

**FIGURE 3:**
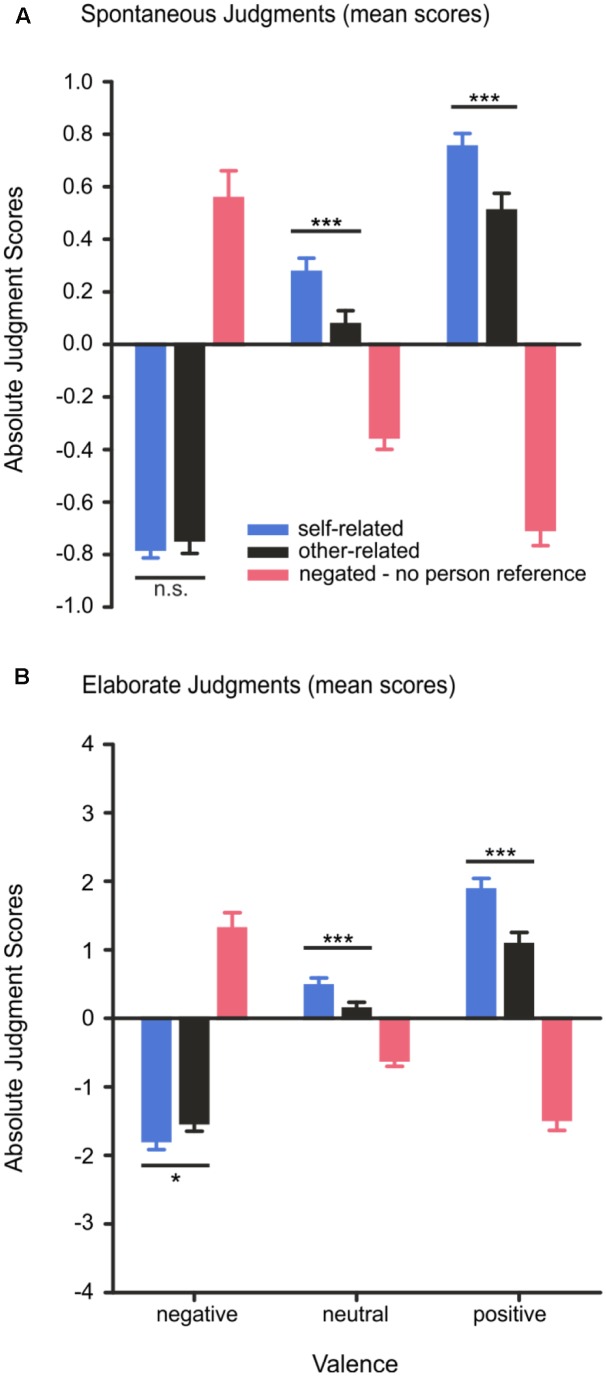
**(A,B)** Spontaneous and elaborate valence judgments. For the spontaneous judgment, subjects had to press one of three buttons corresponding to either negative (–1), neutral (0), or positive (1) valence. In the elaborate judgment, subjects had to verbalize a number on a 9-point scale for their rating to indicate the intensity of their feelings. The number ‘one’ represents very negative, the number ‘five’ neutral and the number ‘nine’ very positive. Error bars depict SEM. n.s. *p* > 0.1, ^∗^*p* < 0.05, ^∗∗∗^*p* < 0.001, FDR corrected.

Spontaneous judgments were modulated by *valence, F*(2,56) = 88.97, *p* < 0.001, η^2^ = 0.76, and *reference*, *F*(2,56) = 19.86, *p* < 0.001, η^2^ = 0.42, and by a significant interaction effect of *valence x reference*, *F*(4,112) = 170.54, *p* < 0.001, η^2^ = 0.86. *Post hoc* tests revealed that positive words were judged more often as positive when they were self-related than when they were other-related, *T*(28) = 4.38, *p* < 0.001. Participants responded also more often with a positive key press to self-related neutral words compared to other-related neutral words, *T*(28) > 4.5, *p* < 0.001. For self- vs. other-related negative words no such response bias could be observed: as shown in **Figure 3A**, participants did not judge self-related negative words more often as negative than other-related negative words, *T*(28) = 1.01, *p* = 0.334. Negated positive words were more often judged as negative compared to negated neutral words, *T*(28) = 6.75, *p* < 0.001, and negated negative words were more often judged as positive than negated neutral words, *T*(28) = 8.46, *p* < 0.001, indicating that participants’ spontaneous emotional judgments were based on the semantics of the words.

Elaborate judgments showed a significant main effect of *valence*, *F*(2,56) = 86.01, *p* < 0.001, η^2^ = 0.75, of reference, *F*(2,56) = 16.97, *p* < 0.001, η^2^ = 0.38, as well as a significant interaction effect of *valence × reference*, *F*(4,112) = 154.27, *p* < 0.001, η^2^ = 0.85. *Post hoc* tests showed that subjective feelings elicited by positive and negative word pairs were judged as more intense, i.e., more positive, *T*(28) = 6.32, *p* < 0.001, or more negative, *T*(28) = 9.85, *p* < 0.001, respectively, compared to neutral word pairs. Moreover, positive feelings elicited by self-related positive words (e.g., “my joy”) were rated higher in intensity than were feelings elicited by other-related positive words (e.g., “his joy”), *T*(28) = 5.24, *p* < 0.001. Feelings elicited by negative words were also rated as significantly higher in intensity when they were related to the self (e.g., “my death”) than when they were other-related (e.g., “his death”), *T*(28) = 2.61, *p* = 0.020, suggesting that self-reference enhances the intensity of subjective feelings for positive and negative words during elaborate judgments. Feelings elicited by negated positive words (e.g., “no joy”) were rated as more negative in intensity than were feelings elicited by negated neutral words, *T*(28) = 6.72, *p* < 0.001. Likewise, feelings elicited by negated unpleasant words (e.g., “no death”) were rated as more positive than were negated neutral words, *T*(28) = 8.90, *p* < 0.001, confirming that the negating pronoun reversed the valence of negative and positive words. Results are depicted in **Figure 3B**. An overview of the behavioral results is provided in **Table 3**.

**Table 3:** Descriptive statistics of spontaneous and elaborate valence judgments (*N* = 29).

Judgment type	Reference	Valence		
		
		Positive	Neutral	Negative
Spontaneous, ratings	Self-related	0.76 (0.24)	0.28 (0.25)	-0.79 (0.14)
	Other-related	0.52 (0.32)	0.08 (0.25)	-0.75 (0.24)
	Negated	-0.71 (0.30)	-0.36 (0.22)	0.56 (0.53)
Spontaneous, reaction time	Self-related	1.83 (0.30)	2.13 (0.37)	1.97 (0.37)
	Other-related	1.99 (0.36)	2.12 (0.38)	1.96 (0.39)
	Negated	1.97 (0.29)	2.13 (0.35)	1.94 (0.32)
Elaborate, ratings	Self-related	6.90 (0.75)	5.50 (0.48)	3.19 (0.57)
	Other-related	6.1 (0.81)	5.16 (0.38)	3.45 (0.53)
	Negated	3.50 (0.73)	4.37 (0.35)	6.33 (1.14)


*Spontaneous ratings range from -1 (negative) to 1 (positive). Spontaneous reaction times are reported in seconds. Elaborate ratings range from 1 (negative) to 9 (positive). Standard deviations of all measures are reported in parentheses.*

### Facial Electromyography

**FIGURE 4:**
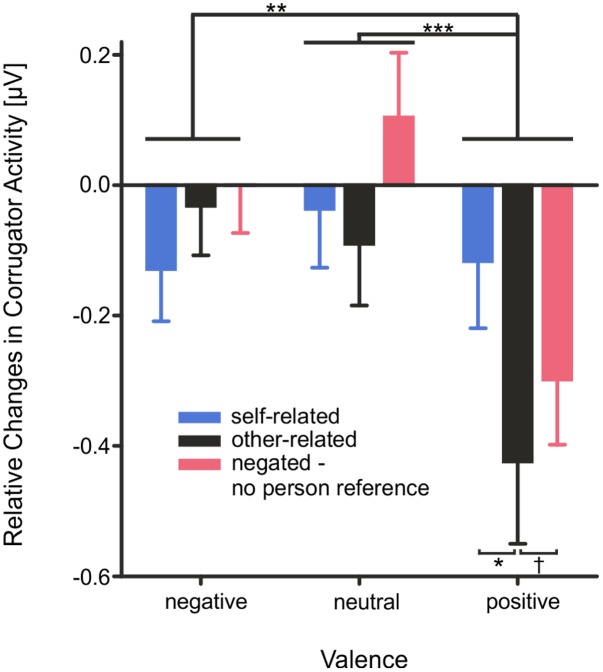
Changes in mean *M. Corrugator* activity as measured by fEMG depicted as changes in mean activity during the stimulus interval from 0 to 4000 ms. Error bars depict SEM. Changes are illustrated as relative changes from baseline. n.s. *p* > 0.1, ^†^*p* = 0.05, ^∗^*p* < 0.05, ^∗∗^*p* < 0.01, ^∗∗∗^*p* < 0.001, FDR corrected.

Mean *M. Corrugator* activity (0–4000 ms, see **Figure 4**) showed a main effect of the factor *valence*, *F*(2,56) = 12.84, *p* < 0.001, η^2^ = 0.31, as well as an interaction of the factors *valence* × *reference, F*(4,112) = 5.75, *p* < 0.001, η^2^ = 0.17. Mean *M. Corrugator* activity was significantly attenuated during the presentation of positive compared to neutral words, *T*(28) = 4.22, *p* < 0.001, or negative words, *T*(28) = 3.55, *p* = 0.002. This attenuation was observed particularly for positive other-related words in comparison to positive self-related words, *T*(28) = 3.67, *p* = 0.002, and trending for other-related positive words in comparison to negated positive words, *T*(28) = 2.08, *p* = 0.050.

**FIGURE 5:**
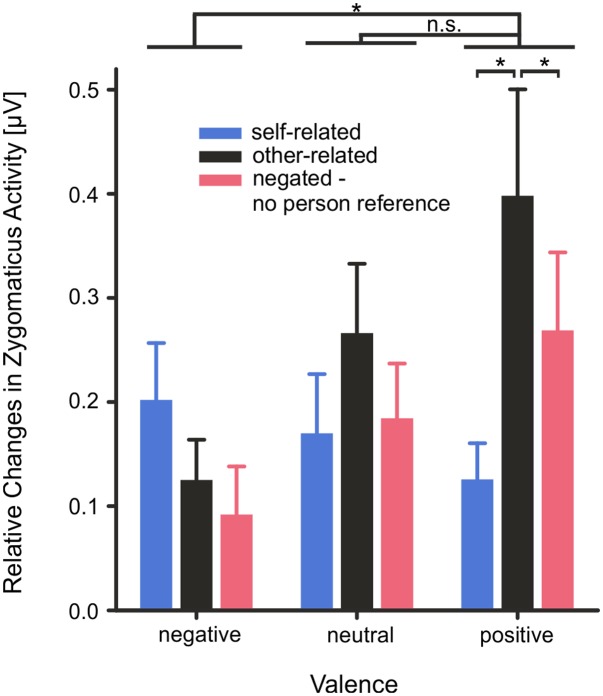
Changes in mean *M. Zygomaticus* activity as measured by fEMG depicted as mean activity during the stimulus interval from 0 to 4000 ms. Error bars depict SEM. Changes are illustrated as relative changes from baseline. n.s. *p* > 0.1, ^∗^*p* < 0.05, FDR corrected.

Mean *M. Zygomaticus* activity (0–4000 ms) showed a significant main effect of *valence*, *F*(2,56) = 4.17, *p* = 0.039, η^2^ = 0.13, and of *reference*, *F*(2,56) = 4.76, *p* = 0.012, η^2^ = 0.15, as well as a significant interaction of *valence × reference*, *F*(4,112) = 3.46, *p* = 0.043, η^2^ = 0.11. Mean *M. Zygomaticus* activity was significantly more pronounced for positive than for negative words, *T*(28) = 2.51, *p* = 0.022. No difference was found between positive and neutral words *T*(28) = 1.11, *p* = 0.278. Also, changes in *Zygomaticus* activity were more pronounced for other-related than for self-related, *T*(28) = 2.48, *p* = 0.023, or negated words, *T*(28) = 2.41, *p* = 0.026. Crucially, changes in mean *Zygomaticus* activity were more pronounced for other-related than for self-related positive words, *T*(28) = 2.52, *p* = 0.022, or negated positive words, *T*(28) = 2.83, *p* = 0.013; see **Figure 5**.

Changes in mean *M. Corrugator* and mean *M. Zygomaticus* activity were not significantly correlated, irrespective of whether self- or other-related words or control stimuli were presented (all *N* = 29, all *p* > 0.1).

### Heart Rate

**FIGURE 6:**
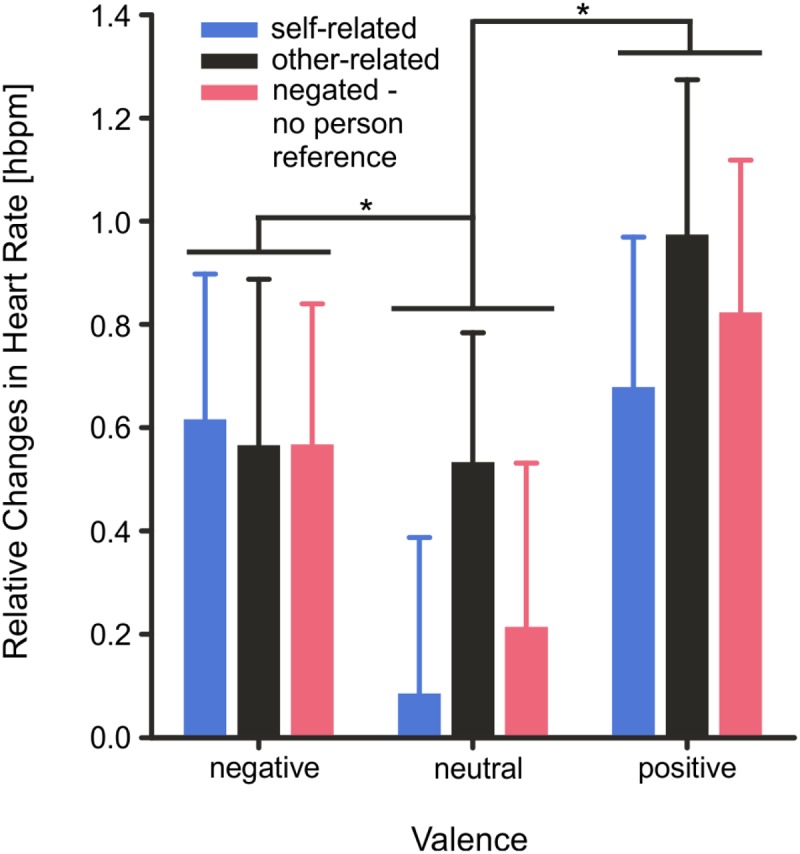
Heart rate activity as measured by 3-Lead ECG and depicted as mean changes during the stimulus interval from 0 to 4000 ms. Error bars depict SEM. Changes are illustrated as relative changes from baseline for all stimulus categories. However, as described in detail in the text, mean heart rate was significantly modulated by the factor *valence* and not by the factor *reference* and the interaction of the factors *valence* × *reference* was not significant. ^∗^*p* < 0.05, FDR corrected.

Changes in mean HR (0–4000 ms) were significantly modulated by the factor *valence, F*(2,56) = 7.99, *p* < 0.001, η^2^ = 0.22, but not by *reference, F*(2,56) = 2.53, *p* = 0.089, η^2^ = 0.08. Mean HR increased significantly during presentation of positive, *T*(28) = 3.57, *p* = 0.002, and negative words, *T*(29) = 2.66, *p* = 0.018, compared to neutral words. The interaction of the factors *valence × reference* was not significant, *F*(4,112) = 0.72, *p* = 0.579, η^2^ = 0.02, (see **Figure 6**).

Analysis of electrodermal activity (exploratory analysis, *N* = 13 subjects) is reported in the Supplement.

### Interindividual Differences

Correlation analyses between behavioral, physiological, and self-reported data (depression, alexithymia, anxiety, and positive and negative affect) revealed no consistent pattern of interactions. Regarding behavioral data, spontaneous judgments of self-related positive words showed a negative correlation with depression (*r* = -0.045, *p* = 0.008, one-tailed) and a positive correlation with self-reported positive affect (*r* = 0.36, *p* = 0.028, one-tailed).

## Discussion

This study investigated reaction times, emotional judgments, and changes in affective physiology, fEMG and HR in particular, during emotional evaluation of words varying in emotional valence and personal reference (self-other reference). Extending previous research supporting an embodied view of language, the present study was aimed at investigating the differential sensitivity of each of these measures to changes in valence (positive, neutral, negative) and personal reference (self, other).

### Behavioral Data (Reaction Time and Judgments)

Participants’ behavioral data indicated preferential processing of positive pronoun-noun phrases, particularly when these were self-related. The preferential processing of self-related positive words was observed during spontaneous judgments and associated with faster reaction times and significantly higher response accuracy. This self-positivity bias was evident when comparing self-related positive words to self-related negative or self-related neutral words and in comparison to other-related positive words as well as control items (i.e., negated words). Elaborate judgments revealed that subjective feelings were significantly more intense when positive and negative words were self-related than when they were other-related.

The self-positivity bias in reaction times is in line with recent EEG studies reporting a processing bias for positive words in designs in which the valence of a word and its personal reference (self-other reference) were experimentally manipulated or controlled for (e.g., Watson et al., 2007; Herbert et al., 2008, 2011b; Fields and Kuperberg, 2012, 2015). The present results confirm these findings on a behavioral level and suggest that participants have faster access to self-related positive information than to self-related negative information in support of mood congruent processing, mildly positive mood being the norm in healthy subjects (Diener et al., 1997; Mezulis et al., 2004). Crucially, the present results attest that it is the self-reference of a stimulus that improves the bias toward positive information, facilitating spontaneous judgments to positive words when their content is related to the reader’s self.

In general, mean reaction times appeared to be slower than reaction times reported in word processing studies using, for instance, lexical decision tasks. However, reaction times greater than one second have been reported in previous studies using emotional evaluation task (see for instance, Niedenthal et al., 2009). Moreover, previous EEG-ERP studies using similar stimulus material as the present one (e.g., Herbert et al., 2011b,d) found a processing advantage for self- versus other-related emotional words specifically at later cortical processing stages in the time windows of the N400 (e.g., Herbert et al., 2011d) or the late positive potential, LPP (e.g., Herbert et al., 2011b; see also Fields and Kuperberg, 2012, 2015). Hence, for abstract stimuli such as words, discrimination between self and other might appear earliest at the level of semantic stimulus integration, and thus temporally after the initial emotional content conveyed by nouns and its personal relatedness (conveyed by pronouns) have been integrated into one semantic concept. Thus, a certain degree of semantic processing is required to discriminate emotional stimuli related to the self from those related to the other. That judgments were based on semantics and thus on the meaning of the word phrases was confirmed by the judgments of the control stimuli: pronoun-noun pairs containing a negation (e.g., “no joy,” “no death”) reversed the direction of the valence judgment for negative and positive words, which is possible only if the negation term is semantically taken into consideration (see e.g., Kaup and Zwaan, 2003; Herbert et al., 2011a).

### Physiological Data [fEMG, HR, and Skin Conductance (EDA)]

Interestingly, despite a processing advantage of self-related positive words in the behavioral and subjective measures, this bias was not accompanied by activity changes in fEMG or HR data. Physiological data did by no means point toward stronger embodiment of self-related positive words in comparison to other-related positive words.

Changes in HR were modulated by the emotional valence of the stimuli with significantly stronger HR acceleration patterns for positive and negative than neutral words during the first 4 s of spontaneous word evaluation. Basic changes in HR in response to the a word’s emotional tone (positive or negative vs. neutral) may occur in anticipation of approach or defense (Bradley and Lang, 2007) and may be larger for emotional stimuli rated higher in emotional arousal than neutral words (Bradley et al., 2008). Of note, the positive and negative nouns chosen for the experiment differed not only in emotional valence but also in emotional arousal from neutral nouns. The observed HR changes therefore fit well with previous reports showing that increases in HR evoked by positive and negative stimuli are modulated by emotional arousal (Bradley et al., 2008) and by preparation for action (Bradley and Lang, 2007).

Mean *M. Zygomaticus* as well as mean *M. Corrugator* activity revealed significantly stronger activity changes during emotional evaluation of positive words. In particular, changes were more pronounced for other-related than self-related positive words. Whereas *M. Zygomaticus* activity showed a significant activity increase, *M. Corrugator* activity showed a significant decrease particularly during the evaluation of other-related positive words as compared to self-related positive words. While activity increases in mean *M. Zygomaticus* activity are reliable indicators of positive emotions, decreases in *M. Corrugator* activity below baseline have also been observed in previous studies in response to positive stimuli eliciting relaxation or surprise (Neta et al., 2009). In the present study, changes in mean activity of the *M. Zygomaticus* and the *M. Corrugator* muscles were not significantly correlated during word evaluation, suggesting that changes in both muscles reflect different facets of emotion processing.

Regarding the processing of concrete emotional stimuli such as faces, response peaks in fEMG have been reported to occur quickly after stimulus presentation (e.g., Dimberg, 1982; Dimberg et al., 2000), which may indicate spontaneous readout of the reader’s own emotions. Regarding previous studies using single emotional words the earliest changes in fEMG activity were occasionally observed as early as 500 ms after word presentation (Foroni and Semin, 2009). However, Niedenthal et al. (2009), for instance, in an emotional evaluation task reported considerably longer latencies. Moreover, previous EEG studies outlined above (e.g., Herbert et al., 2011b; Fields and Kuperberg, 2015) suggest that discrimination between self-related and other-related emotional words may occur during later stimulus processing stages for why changes in facial responses may also occur later for pronoun-noun pairs than for single words. Future research investigating the time course of changes in fEMG during the evaluation of self- vs. other-related emotional words is needed to answer this question (please see the Supplement for a first exploratory and descriptive overview regarding changes in biosignals across the time window of word presentation).

Nevertheless, positive words elicited stronger emotion-congruent changes in mean *M. Zygomaticus* activity when other-related than when self-related. Differential facial responses to positive words support the assumption that people spontaneously and preferentially mimic in relation to others, even if the other is only a virtual other (Fridlund, 1991). In line with this hypothesis and the present observations, it has been suggested that facial expressions as well as feedback from motor and action units of the face are considered particularly important for understanding other people’s actions and emotional states (Rizzolatti and Craighero, 2004; Niedenthal, 2007; Gallese, 2009). The present data might therefore support the view that – as far as verbal input is concerned – facial expressions preferentially occur in response to other-related emotional stimuli; in particular to positive stimuli (Fridlund, 1991), at least when the intention is to evaluate other-related emotional words for their hedonic pleasure. Crucially, participants were not instructed to feel into the emotions of others or empathize with them during evaluation of other-related words, suggesting that it is unlikely that empathy or individual differences in empathy have influenced the results. Personality traits and interindividual differences in mood and affect may modulate facial responsivity (e.g., Ferri et al., 2010). In the present study, participants scoring high in self-report measures of depression or alexithymia were excluded from participation, which reduced the chance of finding strong correlations between these self-report, physiological and behavioral measures. Nevertheless, regarding spontaneous judgments, appraisal of self-related positive words was negatively correlated with depression scores and positively correlated with self-reported positive affect. Although, these correlations do support the hypothesis of mood congruent processing being the cause of the self-positivity bias in emotional judgments in healthy subjects, these correlations should be treated with caution and need to be validated in larger sample sizes.

Taken together, the observed fEMG results could be a challenge for traditional associative network models of language processing (Lang, 1979; Bower, 1981). According to these models, fEMG activity during word reading would be the result of activation spread after memory activation. For instance, activation of the words *happiness* or *joy* would lead to spread of activation to associated concepts (e.g., *smile*), thereby leading to changes in the associated parts of the peripheral nervous system, e.g., the neurons controlling facial musculature (see e.g., Lang, 1979; Bower, 1981). Viewed from this perspective, the fEMG findings would imply that nodes are more strongly interrelated in memory for other-related than for self-related positive information. This conclusion contrasts with the behavioral results as well as with several previous findings predicting overall better memory and prioritized processing for self-related information (self-reference effect; for a meta-study, see Symons and Johnson, 1997).

Differences in cognitive versus affective appraisal strategies could be one reason for differential facial involvement in the evaluation of other- versus self-related emotional words in the present study (e.g., Niedenthal et al., 2009). This speculation is, however, unlikely because the instruction was the same for all words. In addition, in the manipulation check participants did not self-report any processing differences between word categories (self, other, no reference). Thus, possible differences in appraisal strategies (cognitive versus affective) cannot explain why participants “frowned” less and particularly “smiled” more when evaluating other- versus self-related positive words. Moreover, abstractness has been shown to affect the magnitude of facial expressions: for instance, fEMG is larger for emotion-related action words (e.g., smiling, crying, etc.) than for emotional words (e.g., adjectives such as happy, funny etc.; e.g., Foroni and Semin, 2009; Fino et al., 2016). However, abstractness cannot account for the differential effects in fEMG during evaluation of self- and other-related pronoun-noun pairs: words were carefully matched on this dimension and the same set of nouns was presented in each condition such that stimulus-reference was the critical dimension signaling whether nouns were self-related or other-related. However, gender might have played a role as *N* = 24 out of *N* = 29 of the participants were females and physiological signals including fEMG activity has been reported to be more pronounced in women than in men (Greenwald et al., 1989; Bradley et al., 2001). However, previous studies using similar material (Herbert et al., 2011b,d) as well as pronouns to induce self- or other-reference in Western and Asian participant samples (Li and Zhou, 2010; Zhou et al., 2010; Blume and Herbert, 2014) did not report any gender effects. Nevertheless, gender differences should be examined further in future studies using larger sample sizes.

## Conclusion

In the present study, the personal reference (self-other reference) and the emotional valence of words were experimentally manipulated to assess the impact of these dimensions on behavioral, subjective, and physiological responses during an emotional word evaluation task. Whereas behavioral responses indicated preferential processing of self-related positive words, facial responses were most pronounced during evaluation of other-related positive words. Moreover, changes in HR occurred during evaluation of emotional compared to neutral words regardless of their personal reference. Thus, behavioral responses support a self-positivity bias in emotional judgments whereas changes in fEMG seem to support sociality effects (Fridlund, 1991). Physiologically, bodily signals may contribute differently to the emotional evaluation of verbal content with facial expressions, *M. Zygomaticus* activity in particular, being most pronounced during the evaluation of other-related emotional content, positive in particular (Fridlund, 1991), and HR being modulated by differences in emotional content irrespective of whom the information may refer to. Crucially, further studies are needed to scrutinize and validate these assumptions in different settings including actual conversations taking place in both laboratory and real-life settings. The paradigm used in the present study might be especially fruitful to this end.

## Author Contributions

This manuscript is a shared first authorship of PW and CH. CH wrote main parts of the manuscript, designed the study, supervised the study, and analyzed the data together with PW (master student supervised by CH). The study is part of a project funded by the German Research Foundation (HE5880/3-1), awarded to CH. PW helped writing the manuscript including the method section, conducted the study (programmed the design, recorded the data) and analyzed the data together with CH.

## Conflict of Interest Statement

The authors declare that the research was conducted in the absence of any commercial or financial relationships that could be construed as a potential conflict of interest.

## Footnotes

1 http://wortschatz.uni-leipzig.de

2 In accordance with Norman (2010), index scores computed from ordinal items are interval scaled and can be validly analyzed in repeated measurement analyses of variance. It has also been widely argued that ANOVA analyses are robust against violations of the normality assumption that could be induced by ordinal data (e.g., Norman, 2010).
