# Supplementary material for: Bodily Reactions to Emotional Words Referring to Own versus Other People’s Emotions
Source: Front Psychol. 2017 Aug 22;8:1277. doi: 10.3389/fpsyg.2017.01277 (PMC5572286; doi:10.3389/fpsyg.2017.01277)
Supplement: Supplementary file 1 [file Data_Sheet_1.docx]

**Bodily Reactions to Emotional Words Referring to Own versus Other People’s Emotions**

***Supplemental Materials***

Patrick Weis^1*^, Cornelia Herbert^1,2*^

(*both authors contributed equally, joint first authorship)

^1^ Department of Psychiatry, University of Tübingen, Germany

^2^Institute of Psychology and Education, Department of Applied Emotion and Motivation Research, University of Ulm, Ulm, Germany

*First authors (shared first authorship): Patrick Weis and Cornelia Herbert

To be submitted to the Research Topic „The Janus Face of Language: Where are the emotions in words and the words in emotions?“

**Corresponding author:**

Cornelia Herbert

Applied Emotion and Motivation Research

Institute of Psychology and Education, University of Ulm, Germany

Phone: +49-(0)731/50 32850

Fax: +49-(0)731/50 31169

[cornelia.herbert@uni-ulm.de](mailto:cornelia.herbert@uni-ulm.de)


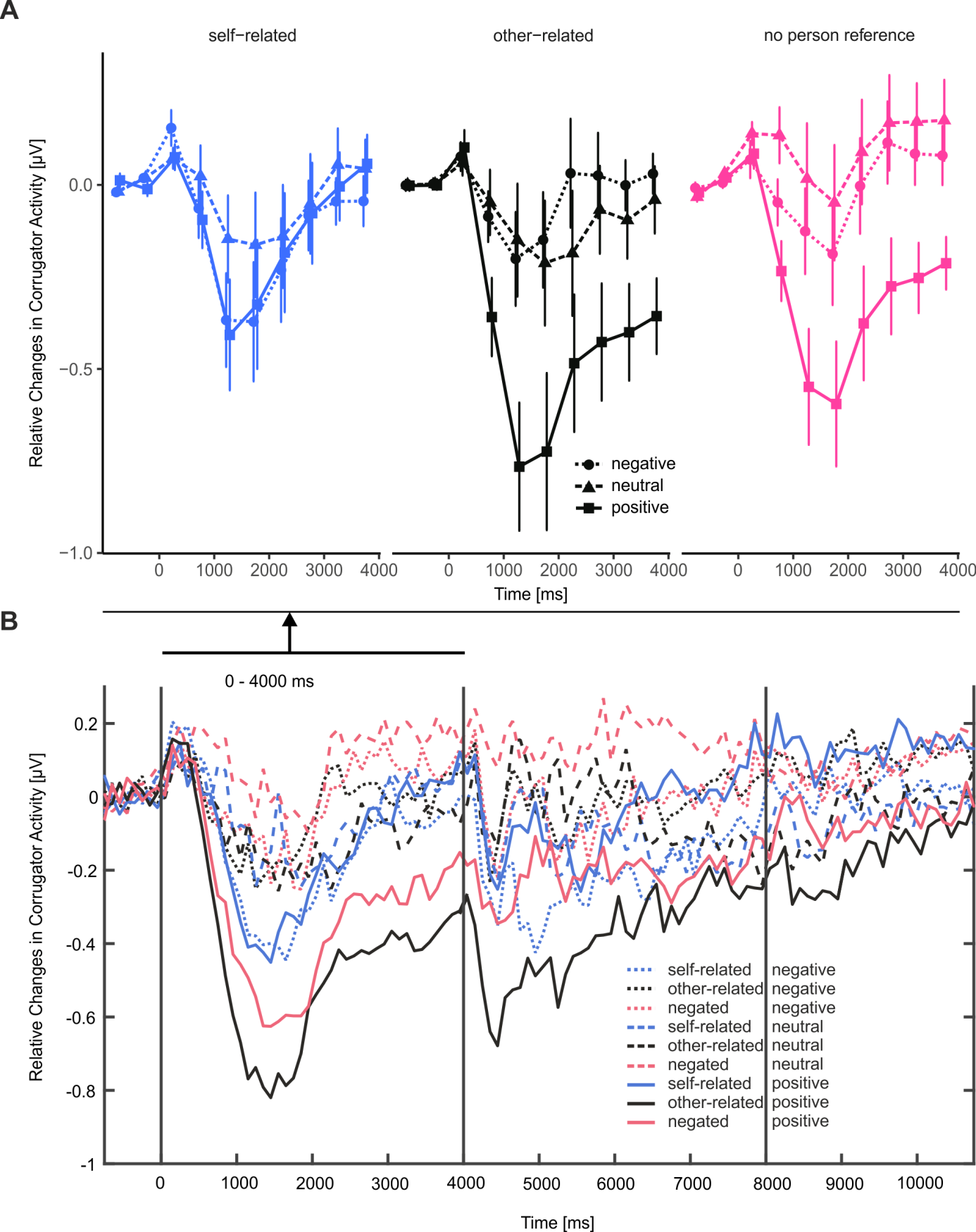


**Figure 1** **(supplement).** *M. Corrugator* activity as measured by fEMG depicted as mean activity changes during the stimulus interval from -1000 ms to 4000 ms after word onset **(A)** or as grand average continuous data from -1000 ms to 11000 ms including the entire trial **(B)**. Time series analysis conducted for the time window from 0 ms until 4000 ms after word onset **(A)** showed that *M. Corrugator* activity varied significantly across *time*, *F*(7,196) = 4.68, *p* = .012, η^2^ = .148. Error bars in **(A)** depict SEM.


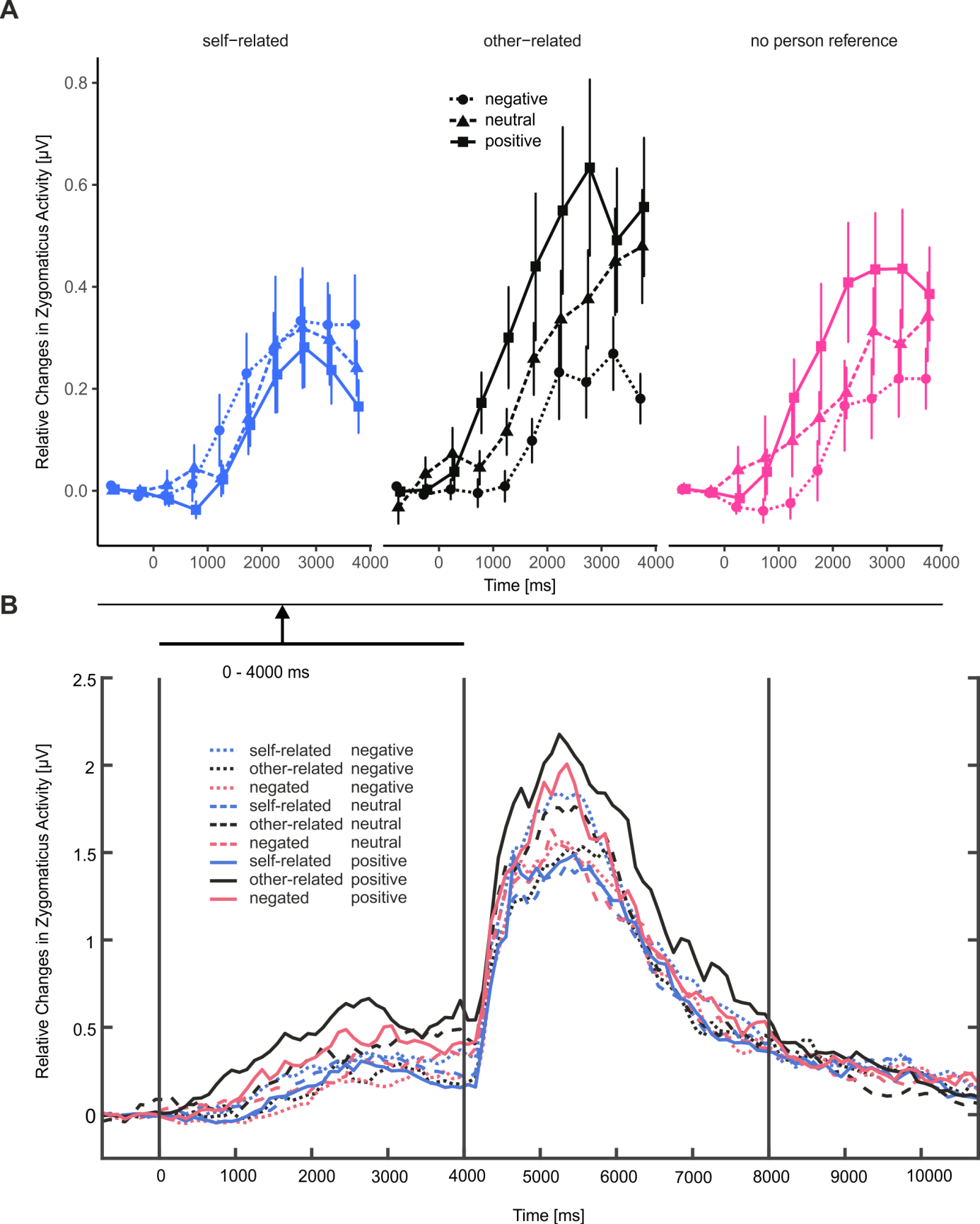


**Figure 2** **(supplement)**. *M. Zygomaticus* activity as measured by fEMG depicted as mean activity changes during the stimulus interval from -1000 ms to 4000 ms after word onset **(A)** or as grand average continuous data **(B)** from -1000 ms to 11000 ms including the entire trial (see Fig. 1). Time series analysis conducted for the time window from 0 ms until 4000 ms after word onset showed that *M*. *Zygomaticus* activity varied significantly across *time*, *F*(7,196) = 17.71, *p* < .001, η^2^ = .387. Error bars in **(A)** depict SEM.


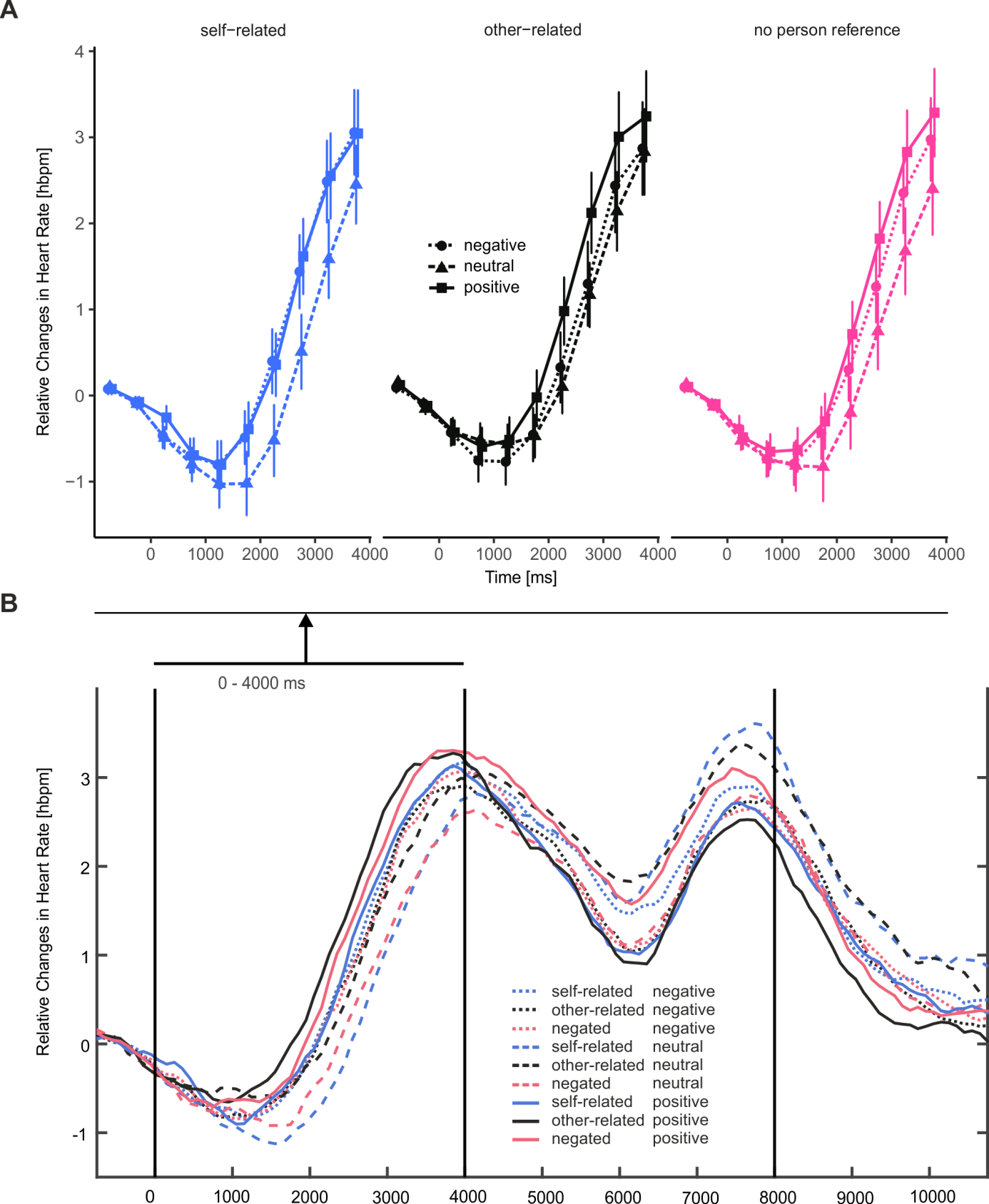


**Figure 3 (supplement).** Heart rate activity as mean changes during the stimulus interval from -1000 ms to 4000 ms after word onset **(A)**, or as continuous data **(B)** from -1000 ms to 11000 ms including the entire trial (see Fig. 1). Time series analysis conducted for the time window from 0 ms until 4000 ms after word onset showed that heart rate varied across *time*, *F*(7,196) = 51.93, *p* < .001, η^2^ = .650. Error bars in **(A)** depict SEM.


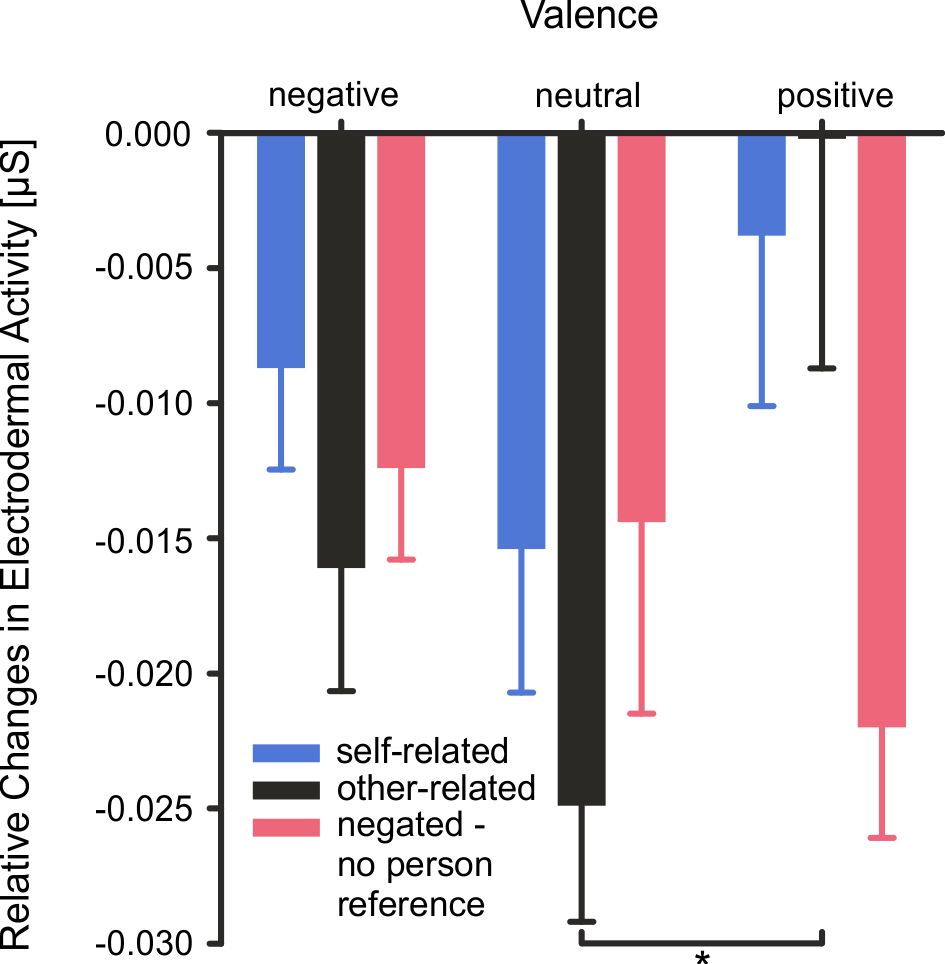


## Figure 4 (supplement). Changes in electrodermal activity (N=13 subjects) depicted as mean activity changes from -1000 ms to 11000 ms after word onset. Error bars depict SEM. Changes are illustrated as relative changes from baseline. An ANOVA revealed that electrodermal activity (0-1100 ms) varied as a function of *valence*, *F*(2,24) = 3.88, *p* = .035, η^2^=.217, but not as a function of *reference* *F*(2,24) = 1.36, *p* = .276, η^2^=.093. The interaction between v*alence* x *reference* was significant*, F*(4,48) = 2.61, *p* = .047, η^2^=0.168, suggesting an increase in activity during processing of self- and other-related positive words. * *p* < .05, FDR corrected.


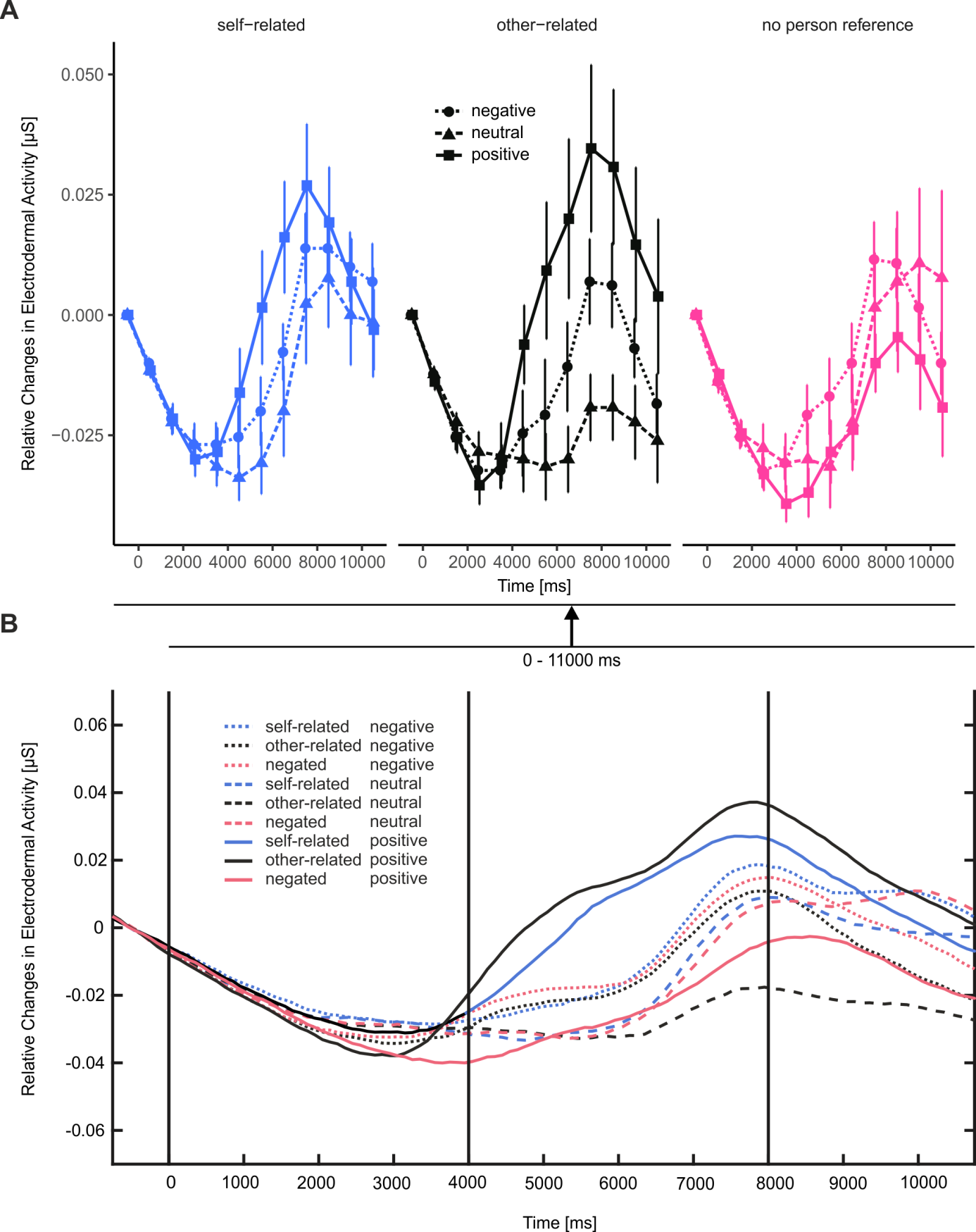


**Figure 5 (supplement).** Changes in electrodermal activity (N=13 subjects) during the interval from -1000 ms to 11000 ms after word onset. Changes are illustrated as relative changes from baseline; **(B)** shows continuous data. Time series analysis conducted for the time window from 0 ms until 11000 ms after word onset showed that EDA varied across *time*, *F*(10,120) = 15.33, *p* < .001, η^2^ = .561. Error bars in **(A)** depict SEM.
